# HSV-1 Infection in Retinal Pigment Epithelial Cells: A Possible Contribution to Age-Related Macular Degeneration

**DOI:** 10.3390/v17081056

**Published:** 2025-07-29

**Authors:** Victoria Belen Ayala-Peña

**Affiliations:** 1 Departamento de Biología, Bioquímica y Farmacia, Universidad Nacional del Sur, Bahía Blanca CP8000, Argentina; vayala@criba.edu.ar; 2 Consejo Nacional de Investigaciones Científicas y Técnicas (CONICET), Buenos Aires CP8000, Argentina

**Keywords:** age-related macular degeneration, amyloid beta peptide, eye, HSV-1, retinal pigmented epithelial cell

## Abstract

Herpes simplex virus type 1 (HSV-1) is associated with eye infections. Specifically, the acute consequences of eye infections have been extensively studied. This review gathers information on possible collateral damage caused by HSV-1 in the retina, such as age-related macular degeneration (AMD), a neurodegenerative disease. The synthesis and accumulation of Amyloid-β peptide (Aβ) is a key hallmark in these types of pathologies. AMD is a disease of multifactorial origin, and viral infections play an important role in its development. It is known that once this virus has entered the eye, it can infect adjacent cells, thus having the ability to infect almost any cell type with great tropism. In the retina, retinal pigment epithelial (RPE) cells are primarily involved in AMD. This work reviews publications that show that RPE can produce Aβ, and once they are infected by HSV-1, the release is promoted. Also, all the information available in the literature that explains how these events may be interconnected has been compiled. This information is valuable when planning new treatments for multifactorial neurodegenerative diseases.

## 1. Introduction

Neurodegenerative diseases are pathologies in which neurons stop functioning or die. Neurodegenerative disorders usually worsen over time and have no effective treatment or cure [1]. They are considered to be multifactorial pathologies. Although the exact causes of neurodegeneration are unknown, factors like aging, genetic risk factors, alcohol intake, tumors, and environmental stressors play a part [2]. However, there is growing evidence that viruses may be linked to neurodegenerative diseases and that early stages of disease development may be impacted by virus-induced neuroinflammation and disruption of neuronal protein quality control [3]. In the eye, the retina has specialized neurons, which can also be damaged during neurodegenerative disease [4]. Age-related macular degeneration (AMD) is one of the neurodegenerative pathologies that affect the eye. One of the most studied examples of neurodegenerative disorders is Alzheimer’s disease (AD), which causes cognitive and motor impairment, primarily affecting the brain [5]. AD and AMD are closely related in terms of their pathophysiology and origin [6,7,8]. The retina is the innermost layer of the eye that shares structural and pathophysiological pathways with the central nervous system, including a connection between the microvasculature and axonal projections [9] which contains a diverse population of neurons [10,11]. The brain and retina share similarities in their structure, development, and the types of pathologies they experience, making them analogous to each other for understanding their pathophysiology. Both structures interact intimately with the adjacent vasculature through the blood–brain and blood–retinal barriers. Furthermore, with increasing age, the retina and the brain develop extracellular deposits associated with degenerative pathology, called drusen and senile plaques, respectively [12]. Peculiarly, AD has been compared to AMD due to the presence of amyloid beta peptide (Aβ) [12,13]. Both pathologies occur with neurological deterioration and have been associated with the particular accumulation of Aβ. Also, in both diseases an association with HSV-1 infections has been observed [14,15,16,17,18,19,20,21]. This work focuses on studying how viral infections are related to the production of Aβ and will compare information on AMD with what has already been described for AD.

## 2. Age-Related Macular Degeneration (AMD)

AMD is one of the most common causes of irreversible blindness, affecting more than 200 million people worldwide, and is expected to affect 288 million people by 2040 [21]. As the leading cause of blindness in people over 55 years old in the United States and Europe, it can also affect younger people. As of right now, AMD has no known cure or viable treatment [1]. AMD is a chronic pathology characterized by a gradual loss of vision due to damage to retinal pigment epithelial (RPE) cells and the subsequent release of inflammatory cytokines and photoreceptor neuron damage [22]. AMD results from a complex interplay between metabolic functions, genetics, and the environment, a milieu that favors long-term structural alterations in the macular area. Local inflammation promotes drusogenesis, RPE and photoreceptor degeneration, brunch membrane disruption, and the development of choroidal neovascularization. Thus, inflammation is believed to play indispensable roles in the pathogenesis of AMD [23]. Since RPE functions as a blood–retinal barrier for the neuroretina and takes part in the maintenance of the photoreceptors, RPE dysfunction has been considered the main participant responsible for AMD progression [24].

Early AMD is often asymptomatic but is characterized by RPE dysfunction and sub-RPE deposits, known as drusen, which are medium-sized and clinically visible [12]. These deposits consist of esterified and unesterified cholesterol, metal ions (iron, zinc), immune cells, RPE cell debris, and different proteins (including complement regulatory factors, apolipoproteins, and Aβ) generally in the macula [12]. Larger drusen and pigmentary anomalies of the macular structure are linked to further disease development in intermediate AMD. Therefore, RPE cells are the initial protagonists involved in AMD pathology. Central vision loss from severe RPE and photoreceptor atrophy (known as “dry AMD”) and/or invasive blood vessel penetration through the blood–retinal barrier (known as “wet AMD”) are the hallmarks of late AMD [12]. Currently, the most prevalent dry form of the disease has no cure; several clinical strategies are used to treat the less common but more aggressive wet AMD, with variable degrees of success [12]. Geographic atrophy (GA) and neovascular AMD are examples of advanced AMD. GA is characterized by progressive retinal thinning caused by the death of photoreceptors, RPE cells, and choroidal capillaries. Neovascular AMD is associated with choroidal neovascularization, an invasion of new choroidal blood vessels into the subretinal and/or sub-RPE area, where fluid exudation could cause blinding [21].

## 3. Amyloid Beta Peptide (Aβ) in AMD

Aβ is a hydrophobic peptide generated through the cleavage of a large protein called amyloid precursor protein (APP), which is a type 1 membrane glycoprotein with important biological functions [25]. Typically, the enzyme BACE1 (β-secretase) produces Aβ in the amyloidogenic pathway, as observed in AD [26]. Another enzyme, called α-secretase, appears to play a predominant role in the non-amyloidogenic pathway [26]. Peculiarly, the activity and expression of BACE1 has been found to be elevated in patients with AD [26]. BACE1 splits APP at amino acid position 671 in the amyloidogenic pathway, releasing sAPP-β and creating a membrane-associated 99-amino-acid C-terminal fragment (C99). Then, γ-secretase sequentially cleaves C99 in several sites, to release several fragments of 43, 45, 46, 48, 49, and 51 amino acids, which are further cleaved to the main final Aβ forms: Aβ40 and Aβ42 [26]. They have both been identified as important peptides in early events in the pathogenesis of AD [26,27].

AMD and AD have been associated with a predominantly inflammatory state, and it is known that Aβ can activate the NF-κB pathway [28,29], related to the activation of proinflammatory cytokines [29,30]. Prior research on AD pathology showed that the p65 component of NF-κB attaches to the κB sites on the BACE1 promoter, causing BACE1 to be expressed [29]. Other studies have shown that proteolytic processing of the amyloid precursor protein is inhibited by inactivation of the NF-κB pathway, resulting in a reduction in Aβ production [31], and that Aβ stimulates NF-κB activation in neurons and glial cells [29]. APP is expressed in several tissues, including those of the retina, and seems to promote synaptogenesis as well as the growth and survival of neurons [32]. Soluble amyloid precursor protein (sAPPα) has a neuroprotective role in the retina and is produced by the proteolytic processing of APP by α- and γ-secretase via the non-amyloidogenic pathway [33].

Aβ accumulation in the retina has been linked with degenerative changes [34]. The predominant alloforms of Aβ in the retina are Aβ42 and Aβ40 [35]. Elevated Aβ levels have been associated with progressive retinal neurodegeneration, especially if deposited in the macula of the retina. The age-related accumulation of such Aβ deposits can be considered as potentially pathogenic reservoirs at critical sites in the retina, contributing to Aβ-mediated “local” chronic toxicity as well as the associated inflammatory events characteristic of degenerative tissues [12].

**Figure 1:**
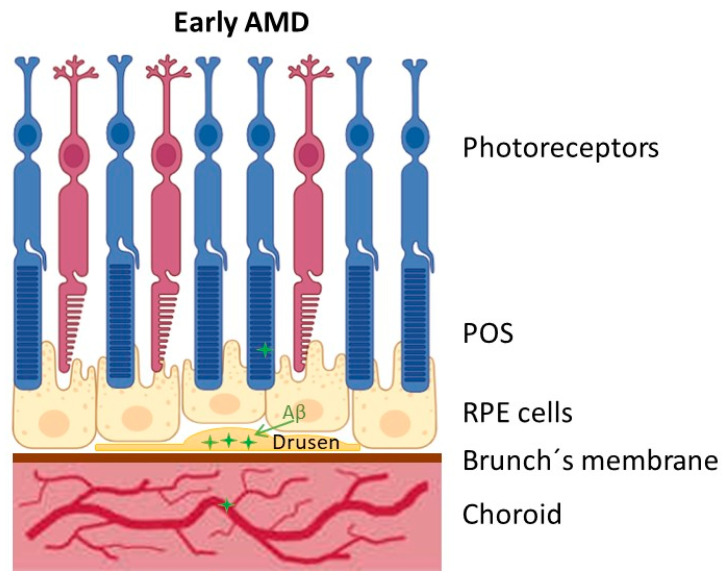
Diagrammatic representation of the accumulation of Aβ (amyloid beta peptide) in the early stages of AMD (age-related macular degeneration). POS: outer segment of photoreceptor; RPE: retinal pigment epithelial cells. The figure was created using some elements from BioRender.com.

APP transcripts and proteins are reported to be abundantly expressed in rat, mouse, and human retinal ganglion cells (RGCs) and RPE cells [12,36,37,38,39]. Also, RPE cells and the RGCs (retinal ganglion cells) of the retina are sources of production and segregation of Aβ. These cells express β-secretase, γ-secretase, and the three main APP isoforms, APP770, APP751, and APP695, and they have the cellular machinery required to produce Aβ [12]. However, RGCs contribute to Aβ release associated with age, while RPE cells contribute constitutively [12]. It was demonstrated that isolated RPE cells from wild-type C57BL/6 mice could synthesize and secrete Aβ, while Aβ expression levels increased in rat RGCs with age [12]. Additionally, Aβ secreted by RGCs can be released into the vitreous humor and subsequently transported to the anterior chamber to accumulate in the drusen, or RPE cells can release Aβ directly into the drusen [12]. All phases of AMD progression have been linked to the buildup of Aβ. However, in early-stage AMD patients, the retina’s structure remains mostly unchanged, and RPE and sub-RPE soft drusen were linked to the existence and accumulation of Aβ in the macular region of the retina [39,40,41,42]. One of the main components of drusen, Aβ peptides, are extracellular deposits that are the first indications of AMD. Fewer reports indicate that Aβ deposition could also be found in photoreceptor outer segments (POSs) and choroidal blood vessels, associated with age [12] (Figure 1). In pathological situations, it has been demonstrated that these Aβ monomers spontaneously assemble inside the retina into dimers, trimers, and oligomers [35]. Aβ oligomers are more neurotoxic than fibrils. They are found in the brain of murine models [43] and in the retina [32].

## 4. RPE-AMD-HSV-1

A wide variety of viruses can infect the eye [44], where the direct role of infectious agents implies the potential infection of various cell types within AMD-related tissues (e.g., RPE cells, endothelial cells, macrophages, and retinal microglia). For example, CMV (cytomegalovirus) can first infect blood cells and reach the eye via hematogenous transmission and then infect the RPE [45]. Thus, infectious pathogens in tissues connected to AMD have been linked to a proinflammatory and proangiogenic state, which may promote the establishment of AMD. Furthermore, mechanisms linked to AMD may also point to the involvement of infectious agents, including oxidative stress, the inflammatory component, and the activation of the alternative complement pathway, with microbial origins. It was observed that in astrocytes and in RPE cells, the downregulation of CD46 (regulatory protein of complement activation, autophagy, and inflammation), among other things, promoted complement hyperactivation favoring the development of AD and AMD, respectively, and that this downregulation could be stimulated by viral infections [46]. Uncontrolled complement hyperactivation can lead to inflammation and cell damage [46]. Other systems include autophagy and mitochondrial dysfunctions, the buildup of lipids, amyloid peptides, zinc, and iron in the drusen, and some related to antimicrobial defense [21]. Larsen reviews work with patients and animals and in vitro, detecting several viruses associated with the progression of AMD, such as HIV (human immunodeficiency virus), HBV (hepatitis B virus), HCV (hepatitis C virus), VZV (varicella-zoster virus), CMV, and HHV-6A [21]. Retinal VZV infection could lead to local chronic inflammation, vasculopathy, and deposits of Aβ in drusen. Also, an increase in Aβ is observed in the plasma of patients with symptoms and diagnoses of acute VZV infection [21].

Herpes simplex virus type I (HSV-1) is an enveloped and double-stranded DNA virus and a member of the *herpesviridae* family, prevalent in ocular infections ranging from mild mucosal ulcers to potentially fatal disorders in immunocompromised individuals [47]. HSV-1 is a neurotropic human virus that infects the majority of the human population and is distributed worldwide, establishing a long-term interaction with the infected host [48]. Blepharitis, conjunctivitis, keratitis, uveitis, and retinitis are common ocular signs of HSV-1 infection [49]. However, some manifestations are not so rapidly visible and have been taken into little account. Corneal nerve degeneration, corneal dendritic cell activity, and alterations in biomechanical characteristics in herpetic keratitis have all been shown using in vivo confocal microscopy [50]. Although it is not the sole causative agent, HSV-1 has been determined as the pathogen most clearly associated with the development of AD [14,15,16,17,18]. It has been proposed that chronic or persistent exposure to HSV-1 (with possible reactivations) could be a risk factor for AD [14,15,16,17]. Also, although viral infections are not a direct cause, their presence, specifically HSV-1, has been linked to the progression of AMD [19,20].

Studies have shown that HSV-1 infection can induce oxidative stress, the production of Aβ, and neuroinflammation [51]. One of the mechanisms described in neuronal cells is that HSV-1 infection results in an alteration in the intracellular redox state, promoting a pro-oxidant state and production of reactive oxygen species (ROS) [52,53,54,55,56]. ROS can be produced by the activation of the NADPH oxidase family of enzymes, which are activated by viral infections such as HSV-1, among other viruses [57]. With respect to this, Kavouras [56] states that HSV-1 requires ROS to replicate efficiently. Studies have shown that oxidative stress can promote Aβ production by increasing the activity of β- and γ-secretase, enzymes that cleave APP to produce Aβ [58,59,60]; in turn, Aβ induces the formation of more ROS, showing a vicious circle between them [61,62]. In addition, HSV-1 infection [63,64] and oxidative stress [63,65,66] could damage to the autophagic machinery, a cellular process responsible for the Aβ elimination, causing its accumulation. Consequently, intracellular production of Aβ increases [17,67]. Finally, whether through one or the other or a combination of several mechanisms, an increase in Aβ occurs, which activates inflammatory processes by activating the NF-κB pathway, which can induce cytokine release [28,30].

Another mechanism described in neuronal cells is that HSV-1 binding to the heparan sulfate proteoaminoglycans produced on the plasma membrane of neurons causes Ca^2+^ signals, which in turn cause APP phosphorylation at Thr668, increasing the activity of β- and γ- secretases [17]. High levels of calcium inside the cell can also turn on GSK3 (glycogen synthase kinase 3), which leads to the phosphorylation of Tau [68] which contributes to AD pathology. In this regard, the association between HSV-1 infection and increased Tau phosphorylation in neuronal cells was recently described [69].

It is also known that neuronal cells [17], RPE cells [70], and fibroblasts [71] produce Aβ in response to HSV-1 infections. Various causes unchain the production of Aβ in neuronal cells, viral infections being one of them [14,15,16,17,18,51]. Regarding its production, in the brain, the Aβ increase after viral infections and has been associated with an inflammatory function and the possible function as a virus sequestrant or as an antimicrobial peptide [72]. Aβ may have an antibacterial function in the brain [73] and retina [21], according to authors.

**Figure 2:**
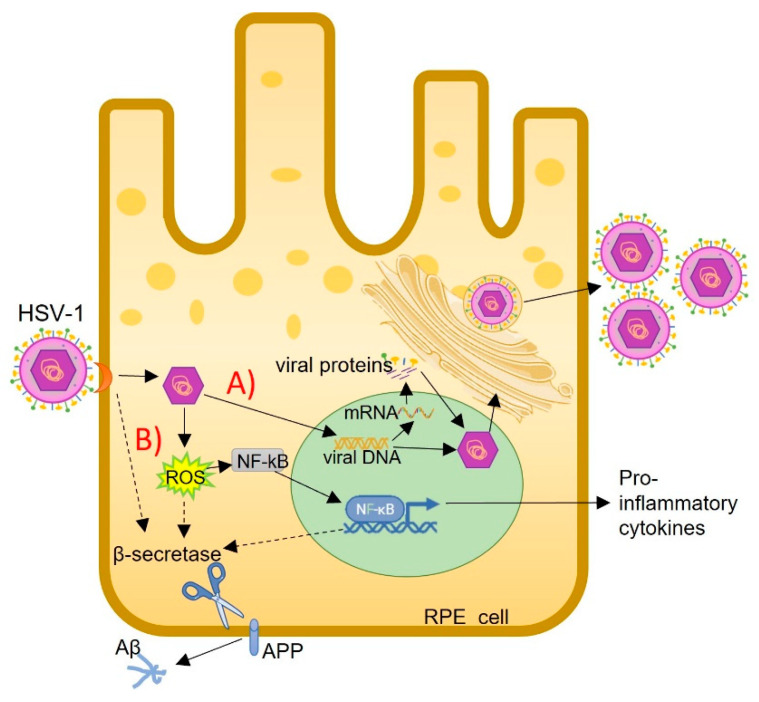
A schematic model of HSV-1 infection in the RPE. (A) The viral replication cycle. (B) The viral infection induces ROS (reactive oxygen species). ROS promote NF-κB and β-secretase activation. NF-κB translocation to the nucleus induces IL-6 synthesis and increases the synthesis of β-secretase. This enzyme cleaves APP (amyloid peptide), and Aβ is released. HSV-1 replicates in the RPE cell, and then the viruses are released. Solid line: pathway present in RPE. Dashed line: possible pathway for β-secretase activation.

Once HSV-1 has infected the RPE, it must carry out its viral replication cycle. It is accepted that HSV-1 replicates in the RPE following the steps already described for other epithelial cells [74,75]. But, in addition to replicating, HSV-1 produces other issues. HSV-1 infection in RPE cells generates ROS [70] (Figure 2), which is not a minor detail, given that RPE cells are particularly susceptible to oxidative stress [76]. When exposed to oxidative stress, the RPE can begin to degenerate into AMD [77,78]. Oxidative stress can generate free radicals that induce various alterations, for example, the oxidation of products such as cholesterol. In this regard, a study shows that oxysterols can increase the production and accumulation of Aβ in RPE cells [79,80] and in neuroblastoma cells [81]. Also, Aβ induces the ROS production in the RPE [82], demonstrating a loop between them. To respect, following infection of the RPE with HSV-1, APP cleavage and Aβ release increase [70]. However, still, there is no data on whether this increase in cleavage is mediated by ROS, an increase in intracellular Ca^2+^, or both in RPE cells. Nevertheless, it should be noted that HSV-1 induces an increase in intracellular calcium, which is required for the penetration step into epithelial cells [83]. In this regard, it is known that the increase in intracellular calcium, and thus the disruption of its homeostasis, is one of the factors that contributes to the accumulation of Aβ in ARPE-19 cells [84]. It was also described that in RPE cells, viral infections, such as by CMV, could induce a decrease in autophagy [85] and that HSV-1 virus infections in the eye disrupt autophagy [86]. Based on the concept that autophagy is a cellular process responsible for Aβ elimination, it is presumable that in this system, its disruption could also cause Aβ accumulation. For all the above, it can be said that, either in one way or by a combination of various mechanisms, in RPE cells, HSV-1 infection also induces the production and/or accumulation of Aβ.

In the RPE, HSV-1 infection induces the activation of NF-κB [70]. Although HSV-1 can activate the NF-κB pathway by signal transduction from virus binding to the membrane receptors HVEM (host receptor herpesvirus entry mediator) and/or TLR2 (toll-like receptor 2) and then activate the NF-κB signaling pathway [87,88,89], this mechanism has not yet been studied in the RPE. As previously stated, NF-κB activation promotes the synthesis of proinflammatory cytokines. In this regard, studies have shown that in the RPE, HSV-1 infection increases the synthesis of IL-6 [70], IL-17A, IL-23 [90], IL1β, IL-18 [91], IL-6, and TNFα [92].

## 5. How HSV-1 Reaches and Infects the RPE

Evidence has shown that HSV-1 can infect RPEs in vitro and in vivo [90,91,93,94,95,96,97,98,99,100,101] but it is important to know how this virus reaches the RPE. Viruses can infect the brain through different routes: ocular, intranasal, and digestive routes, direct inoculation, etc. [102]. Generally, neurotropic viruses (such as HSV-1) reach the brain through neurons [68]. It has been hypothesized that there is a relationship between HSV-1 and AD; in the primary infection, HSV-1 infects the eyes, nose, and mouth, and then, employing cranial nerves, axonal transport occurs retrogradely into the brain [68,103,104]. Comparatively, in a primary ocular infection by HSV-1, this virus can infect the cornea and epithelium, where the virus can cause symptoms and/or replicate and spread to adjacent cells [74,100,104]. Although it is postulated that viruses can infect various cell layers by spreading adjacently from cell to cell, to date there are no experimental studies that demonstrate HSV-1 dissemination from the cornea to the RPE. HSV-1 can infect corneal afferent neurons and travel to trigeminal ganglion by retrograde transport [74,105,106], and then the virus can return to the sites of primary infection during reactivations [74,104,107].

In patients with retinitis, the presence of HSV-1 antigens was observed in all layers of the retina, including the RPE [108]. In vitro tests showed that when infection was induced in the vitreous humor of animals, HSV-1 first infected the axons of the ganglion cells and then spread through all layers of the retina, reaching the RPE [109]. Dogrammatzi and coworkers, in a review of the literature, explains that HSV-1 requires VP22 to spread cell-to-cell [74]. In turn, HSV-1 can infect the neurons of the retina and then move retrogradely towards the cornea or anterogradely towards the CNS, helped by viral gE/gI [74].

**Figure 3:**
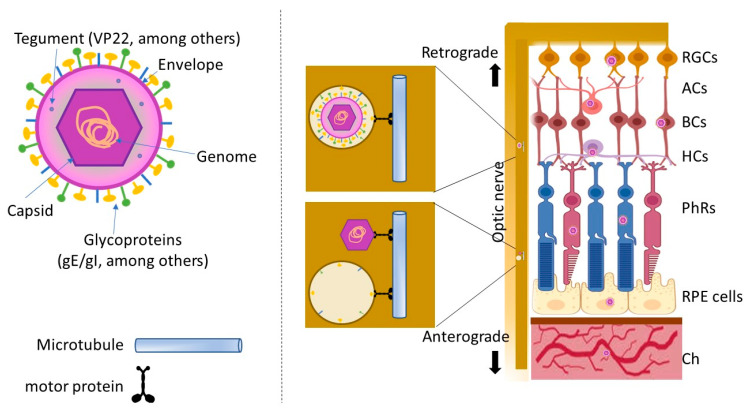
Schematic model of HSV-1 infection in the retina. RGCs: retinal ganglion cells. ACs: amacrine cells. BCs: bipolar cells. HCs: horizontal cells. PhRs: photoreceptor cells. RPE cells: retinal pigment epithelial cells. Ch: choroid. This figure was created using some elements from BioRender.com.

In herpetic encephalitis, reactivated HSV-1 can cause relapses and even spread to the retina, where it can induce a potentially blinding ocular disease known as acute retinal necrosis [110,111,112]. In one study, intraocular inoculation of mice with HSV-1 resulted in brain infection and subsequent retinitis in the fellow eye. The virus was shown to reach the retina of the fellow eye by transaxonal spread through the optic nerve [113]. The elemental viral molecules involved in axonal transport are gE/gI glycoproteins, whether viruses travel as married or divorced mobilized by motor proteins, such as kinesin and dynein superfamily proteins [74,114] (Figure 3). This shows that viruses could also share entry pathways to the brain or retina, which also suggests a possible physical interconnection between AD and AMD. Retrograde or anterograde neuronal transport is probably the most common; nonetheless, we must not forget that viruses can infect the eye from other parts as well [100]. The retina and choroid are richly vascularized structures, so they can be colonized by germs through the hematogenous route in the course of a systemic infectious disease and infect the eye by crossing the epithelial barrier [115]. For example, infectious uveitis has been reported to often spread hematogenously from one part of the body to the vascular uvea, where it disrupts the blood–retinal barrier. Early viral infection leads to endothelial cell damage, loss of tight junctions, and thickening of the basement membrane, resulting in increased vascular permeability near the RPE [116]. For example, retinal infection by Zika [117,118] or CMV [119] has been detected coinciding with peaks of viremia. Although it cannot be ruled out, the hematogenous route of HSV-1 infection has not yet been described.

Three families of molecules are known be the main ones to facilitate HSV-1 entry into cells, HVEM (host receptor herpesvirus entry mediator), nectins, and heparan sulfate proteoglycans [104], while others have been described depending on the cell type. In this regard, it has been described that RPE possesses HVEM [54], nectin-1 [87,98], and heparan sulfate [120,121], necessary for HSV-1 infection. To facilitate entry, HSV-1 can use the mannose 6-phosphate/insulin-like growth factor II receptor [96]. It is considered that the greatest viral entry into RPE cells is through the apical face, since to enter through the basolateral face, viruses would need the hematogenous route, and for HSV-1, being a neurotropic virus, entry through the blood–retinal barrier has been poorly described [94].

## 6. Conclusions and Future Perspective

This work presents strong similarity between the neurodegenerative pathologies AMD and AD and their connection with viral infections. This study shows that in AMD, RPE cells are the main cell type involved in Aβ production, and it shows how HSV-1 can reach the RPE to infect, produce Aβ, and induce inflammation. Several mechanisms triggered by viral infections are shared by AD and AMD, such as the increase in and accumulation of Aβ, increased intercellular calcium, increased ROS, NF-κB activation, and inflammation. Although several studies have demonstrated this similarity, to complete our understanding, future studies should focus on further elucidating more deeply each of the molecular mechanisms involved and, thus, develop antiviral drugs that can also modulate Aβ production. It is important to consider the routine screening of HSV-1 infection in patients with AMD so that specific treatment can be applied. Some studies involve viral serodiagnosis in patients with AMD, because this is a non-invasive technique. For example, for HSV-1 [20], or CMV [122], it was seen that patients with AMD had higher titers of specific antibodies than control patients. Regarding treatments for neurodegenerative diseases associated with viral infections, acyclovir and its analogs have been tested in clinical trials for AD treatment in patients infected with HSV-1, with encouraging results [123]. In this stage, it is important to look into whether possible treatments for AD could also work for AMD and to think about suggesting combined therapies for treating or preventing neurodegenerative diseases linked to viral infections.

## Data Availability

No data were used for the research described in this article.

## Abbreviations

The following abbreviations are used in this manuscript:

Aβ	Amyloid-β peptide
AD	Alzheimer’s disease
AMD	Age-related macular degeneration
APP	Amyloid precursor protein
CNS	Central nervous system
CMV	Cytomegalovirus
GA	Geographic atrophy
HBV	Hepatitis B virus
HCV	Hepatitis C virus
HIV	Human immunodeficiency virus
HSV-1	Herpes simplex virus 1
IL	Interleukin
RGCs	Retinal ganglion cells
POS	Photoreceptor outer segment
ROS	Reactive oxygen species
RPE	Retinal pigment epithelium
VZV	Varicella-zoster virus
